# Stress effects on food handlers’ attention of a public hospital in Recife-PE, Brazil

**DOI:** 10.1590/S1980-57642010DN40400012

**Published:** 2010

**Authors:** Renata de Melo Guerra Ribas, Valdenilson Ribeiro Ribas, Hugo André de Lima Martins, Valéria Ribeiro Ribas, Severino Marcos de Oliveira Carneiro, Rosângela Nieto de Albuquerque, Paulo Henrique da Silva Andrade, Ana Cristina de Melo Guerra, Luciano da Fonseca Lins, Marisilda de Almeida Ribeiro

**Affiliations:** 1 Expert Sanitary Surveillance; 2 PhD in Food Science; 3 PhD in Neuropsychiatry; 4 Master in Neurology; 5 Master in Neuroscience; 6 Master Student in Educational Psychology; 7 Master in Language Sciences, Catholic University of Pernambuco; 8 Graduated in Physical Education; 9 Graduated in Business Administration; 10 Post-Doctorate in Science Education

**Keywords:** stress, attention, food handlers

## Abstract

Production of meals in Food and Nutrition Units (FNU) requires human labor performance in order to attend demand. Stress is one of the factors responsible for alterations in the health of these professionals. Reduced attention is one of the negative effects caused by stress.

**Objective:**

The objective of this study was to evaluate attention in Food Handlers (FH).

**Methods:**

67 professionals were evaluated, subdivided into FH with less than 5 years in the profession, who were included as the control group (Control, n=29), and FH with more than 5 years in the profession (FH>5, n=38). Lipp’s Inventory of Stress Symptoms for adults (LISS), the digit symbol, d2, forward digit span, backward digit span and paced auditory serial addition tests were applied. The data found on the LISS were analysed by Fisher’s Exact and Chi-Square tests and individual attention tests were analyzed by the Mann-Whitney test, with data expressed as Median (Minimum and Maximum) with p<0.05. A total of 73.68% of FH>5 presented stress versus 13.79% of Control. Also, 57.89% of FH>5 with stress presented physical symptoms and 15.79% psychological symptoms. In the Control, 9.68% presented physical symptoms.

**Results:**

73.68% of FH>5 with stress and 17.24% of the Control were in the resistance phase. Regarding attention, the FH>5 presented reduced focused attention (32.5±2.9), auditory attention (7.25±0.4) and resistance to interference (4.67±1.1), compared to their respective controls (43.88±1.39), (8.63±0.38), (9.25±1.16), p<0.05.

**Discussion and Conclusions:**

The activity of food handling can cause stress and attention level reduction after 5 years.

## Abstract (pt)

A produção de refeições em Unidades de Alimentação e Nutrição (UAN) dispõe de apoio do desempenho humano. O estresse é um dos fatores responsáveis por alterações na saúde desses profissionais. Redução da atenção é um dos prejuízos causados pelo estresse.

**Objetivo:**

O objetivo deste trabalho foi avaliar a atenção em manipuladores de alimentos (MA).

**Métodos:**

Foram avaliados 67 profissionais subdivididos em MA com menos de cinco anos na profissão que foram considerados grupo de controle (Controle, n=29) e MA com mais de 5 anos na profissão (MA>5, n=38). Foram utilizados o Inventário de Sintomas de Stress em Adultos de Lipp (ISSL), os testes de atenção *digit symbol*, d2, *span* de dígitos em ordem direta e inversa e teste de dependência auditiva para adição em série. Utilizou-se o Exato de Fisher, Qui-quadrado e Mann-Whitney, dados expressos em percentuais e mediana (mínimo-máximo), p<0,05.

**Resultados:**

Verificou-se que 73,68% dos MA>5, enquanto 13,79% do controle apresentam estresse. 57,89% dos MA>5 com estresse apresentaram sintomas físicos e 15,79% sintomas psicológicos. No controle, 9,68% apresentaram sintomas físicos. 73,68% dos MA>5 com estresse e 17,24% do controle estão na fase de resistência. Em relação à atenção, Os MA>5 apresentaram redução do foco de atenção (32,5±2,9), da atenção auditiva (7,25±0,4) e da resistência à interferência (4,67±1,1), comparados aos seus respectivos controles (43,88±1,39), (8,63±0,38), (9,25±1,16), p<0,05.

**Discussão e Conclusões:**

A atividade de manipulação de alimentos pode causar estresse e redução da atenção após 5 anos.

Although the production of meals in Food and Nutrition Units (UAN) relies on technological support to attend demand, the quality of meals is still directly related to performance of human labor.^1^

In any sector of activity, it is important that food handlers operate under physical and healthy working conditions which allow the integration and harmonization of the body and mind, and full use of human potential in carrying out work activities.^2^

These conditions have been shown as a cause of concern and a focus of investigation by researchers who have demonstrated and alerted to the association of physical condition of food handlers and inappropriate work environments in relation to worker health and quality of manufactured food.^3^

Inappropriate conditions of noise, temperature, humidity, lighting, environment and ergonomics, such as repetitive rhythms raise concerns over compromise to the health and work of these food handlers. The nutritional status of overweight is considered to be caused partly by environmental factors and also represents a danger to health, causing vertebral spine overload, leading individuals to inactiveness and chronic stress.^4,5^

Stress is one of the factors responsible for alterations in health and well-being of individuals, causing diseases or even death. This problem is highly expensive, since it is the cause of several diseases and reduces productivity, increasing the rate of absenteeism and compromising the quality of life of these handlers.^5-7^

Emotional stress is a complex and global reaction by the organism that involves physical, psychological, mental and hormonal components and develops in stages or phases. The manifestation of stress may occur in any person, for all human beings are subjected to an excess of stressful factors that surpass their physical and emotional capacity to resist to them. Stress has long-thought to develop in three phases: alarm, resistance and exhaustion; designated as the three-phase model by Selye in 1936. Recently, after 15 years of research at the Stress Laboratory (LEPS) of PUC-Campinas, Lipp identified another phase of stress, designated ‘near-exhaustion’ that lies between resistance and exhaustion stages. Thus, a four-phase model for stress was proposed. In the process of development, it is necessary to consider the symptomatological condition, which may vary depending on the phase in which it is found. In the alarm phase, considered the positive phase of stress, the individual is energized through the production of adrenaline relating to the survival response. In a second phase, considered the resistance phase, the individual automatically interacts with their stressors in order to maintain their internal homeostasis. If stressful factors persist in frequency and intensity, the phase of resistance may evolve to the near-exhaustion phase. At this stage the disease process begins. If there is no relief from stress by removing the stressors or by using coping strategies, the stress reaches its final phase, exhaustion, considered the point at which other pathologies may set in.^8^

Concerning the harm caused by stress, some studies have demonstrated alterations in cognitive performance such as concentration and attention.^9^

Attention has been a topic of concern for studies on the production area of nutrition, because a food handler who presents attention deficit may make errors in food manufacturing or cause an accident in the kitchens.

Several authors have proposed theories related to the functioning of attention. However, Mateer & Mapou (1996) presented a model that integrates all the theories proposed. These authors proposed that attention be divided into two cognitive factors: deployment and encoding.^10^

Deployment is the ability to channel attention for specific stimuli and maintain the attention on this stimulus.^11^

Encoding is the individual’s ability to store information in memory, in the short term, and mentally manipulate this information.^12^

The combining of these abilities shows the dimension of human attention in its plain functioning with characteristics that include level of alertness, focused attention, sustained attention, information storage, mental manipulation and resistance to interference. However, these abilities may be compromised if there is interference by variables such as stress.

Although there are studies demonstrating the presence of stress in the life of food handlers, there are no studies available in the literature investigating the cognitive harm in cases of reduced attention performance of these workers.

Thus, this study aimed to investigate whether stress in these food handlers or the occurrence of stress, led to a reduction in the attention level of these individuals.

## Methods

### Subjects

The food handlers were randomly selected from the Food and Nutrition Unit of the Public Hospital in Recife, Pernambuco, Brazil. The sample comprised 67 male individuals. All subjects had a basic education, and were receiving a minimum wage. The subjects of similar age within the parameters established (20-35 age group) were divided into 2 groups: [a] Food Handlers with less than 5 years in the profession (Control, n=29) and b) Food handlers with more than 5 years in the profession (FH>5, n=38). The criterion for the group five-year split was established after observation of the employee medical dismissal records in incident report logs, showing an increase in absenteeism among employees of this food and nutrition unit after a 5-year period. It is noteworthy that these professionals received a minimum wage and had no career plan. The study was approved by the Ethics Committee in Research of the Agamenon Magalhães Hospital, in a meeting on June 25 2008, under the rules for research involving human beings (Resolution 196/96). The subjects were submitted to the evaluations under standard conditions at 08:00 a.m. at the beginning of the service day, within an air-conditioned room at a temperature of 22°±2°C. The professionals were informed about the application of the test the previous day and all subjects had agreed to sleep at 08:00 p.m., the day prior to the attention tests. Male Food Handlers were included in the study but female Food Handlers were excluded because of their low number. All tests used in this study were validated and approved for clinical evaluation by the Federal Council of Psychology, except for the PASAT. However, there was no impediment to comparative research using the PASAT.

### Stress evaluation

Stress was assessed by Lipp’s Inventory of Stress Symptoms for adult application (LISS). Easy, practical and fast to apply, the LISS provides an objective measure of stress symptoms in teenagers over 15 years old and adults, since it allows the evaluator to read the items of the questionnaire, and thus does not depend on the literacy of the subject.

The test includes issues related to physical and psychological symptoms of stress within the past month and the last 24 hours of its application. The instrument classifies the symptoms into four phases: alarm, resistance, near-exhaustion and exhaustion. For each phase there is a cutoff, which is checked by adding it to the items that were marked by the subject classified into one of the four phases of stress. After items have been classified, their predominance of physical or psychological symptoms is checked.

### Attention evaluation

The attention assessment was performed by psychologist, Dr. Valdenilson Ribeiro Ribas, medical registration no. CRP 11.797, in compliance with the Brazilian Federal Council of Psychology guidelines. The Federal Council of Psychology previously validated the d2 test by Brickenkamp and Bittencourt in 2000^13^ whereas the digit symbol, digit span forward and inverse tests were validated for the Brazilian population by Nascimento in 2004.^14^ Most tests show standards for scores assessed according to the patient community as sensitive to brain damage, dementia, age and depression etc. Therefore evaluators need to consult a manual, and reference the scores previously validated in published scientific papers.

#### Deployment factor - digit symbol and d2 test application

The digit symbol test requires the correct correspondence of the numbers from 1 to 9 and their respective symbols within 90 or 120 seconds. The digit symbol test contains nine symbols that must be distributed into 130 quadrants according to the corresponding number, with the first seven quadrants reserved for training. The standard for the digit symbol test scores depends on the community assessed. For example, Scores on the Digit Symbol Substitution Test (DSST Denver School of Science and Technology - Colorado charter school) in the study with title cognition, may improve with long-term opioids for non-cancer pain (Stable Doses for Chronic Low Back Pain) which improved from 59.0 at baseline to 64.4 and 65.1 after 90 and 180 days, respectively. The scores represent the total number of correct pairings a patient is able to make within 2 minutes when presented with a series of numbers and corresponding symbols;^15^ while in the d2 the individual must mark the letter d on a specific form containing 14 test lines with 47 letters on each, while maintaining focus on d2, according to the previous explanation, where the position of all letter “d can be differentiated with single or double lines, above or below the letter.

Attention capacity factor - Digit Span Forward – Backwards Digit Span and Paced Auditory Serial Addition Test (PASAT).

**Forward Digit Span** – Commences with a sequence of 2 digits and then increases progressively. The subject must correctly repeat the sequence. This provides an indirect form of measuring the amount of information that the subject is able to retain.

**Backwards Digit span** - Similar to the Forward Digit span only the subject has to repeat the digits sequence in reverse order. For example, given 2-6-8, the individual must repeat as 8-6-2, and so forth. The digit sequences increase in length and the subject keeps repeating in reverse order. Besides maintaining information in the memory, the subject must also be able to mentally manipulate the information.

**PASAT** - This test verifies the mental manipulation capacity of information in addition to the capacity to resist interference. This resistance to interference is the third element that must be evaluated to verify attention capacity. In this test, the subject has to add a dictated sequence of numbers. The examiner states the first number followed by the second and, using this second number, the subject has to add it to the previous one. For example, 4, 7=11. When the next number is uttered, the subject must add this to the last number of the previous sequence, in this case 7, and not with its sum, which was 11. Subjects must then be able to maintain the new number and discard the previous sum. This test proves the subject’s mental manipulation capacity of information and resistance to interference. In this study, the resistance to interference was not evaluated.

### Data analysis

The results found on the Lipp’s Inventory of Stress Symptoms for adults were analysed by Fisher’s Exact and Chi-Square tests, and individually applied attention tests were analyzed by the Mann-Whitney and data expressed as Median (Minimum and Maximum) with p<0.05.

## Results

Stress was present in 73.68% of FH>5 and in 13.79% of the Control (Table 1).

**Table 1:** Presence of stress.

Subjects	Stress			No stress	
	Freq.	%		Freq.	%
Control	4	**13.79%**		25	**86.21%**
FH>5	28	**73.68%**		10	**26.32%**
Total	32			35	

Sixty-seven (67) male food handlers aged 20-35 years. Subjects were divided into 2 groups: food handlers with less than 5 years in the profession (Control, n=29) and food handlers with more than 5 years in the profession (FH>5, n=38). Statistics: Fisher's exact test, p<0.0001.

Physical symptoms were present in 57.89% and psychological symptoms in 15.79% of FH>5 with stress. In the Control, 9.68% presented physical symptoms (Table 2).

**Table 2:** Physical or psychological prevalence.

Subjects	Physical			Psychological			Physical & Psychological			Absence	
	Freq.	%		Freq.	%		Freq.	%		Freq.	%
Control	3	**9.68%**		0	**0%**		3	**9.68%**		25	**89.29%**
FH>5	22	**57.89%**		6	**15.79%**		28	**73.68%**		10	**26.32%**
Total	25			6			31			35	

Sixty-seven (67) male food handlers aged 20-35 years. The subjects were divided into 2 groups: food handlers with less than 5 years in the profession (Control, n=29) and food handlers with more than 5 years in the profession (FH>5, n=38). Statistics: Chi-square test, p<0.0001.

Regarding phases of stress, 73.68% of FH>5 with stress were in the resistance phase, while 17.24% of the Control were in this phase (Table 2).

**Figure 1:**
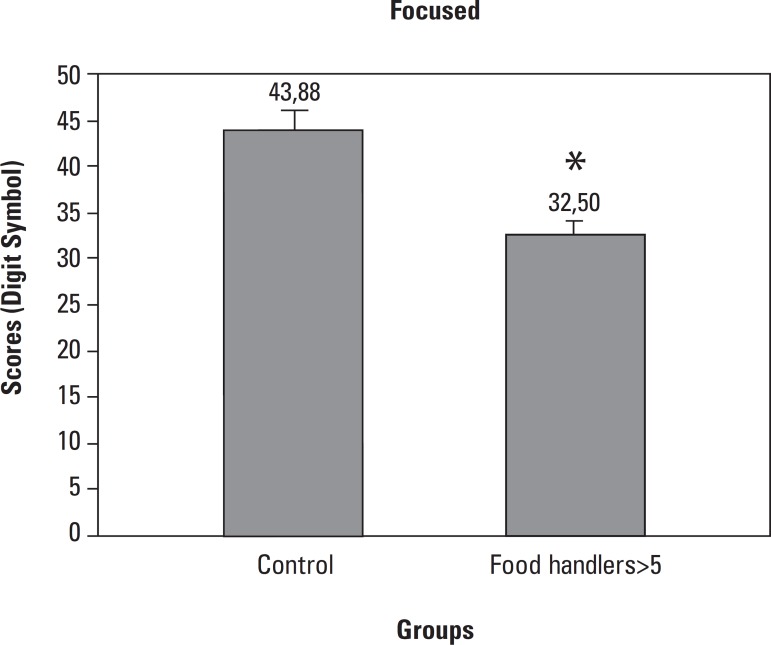
Focused attention was evaluated by the Digit Symbol test in Food Handlers with more than 5 years in the profession (FH>5, n=38) and compared to Food Handlers with less than 5 years in the profession (Control, n=29). Data analyzed by the Mann-Whitney test expressed as Median (Minimum and Maximum), p<0.05*.

In the digit symbol test, FH>5 presented lower focused attention (32.5±2.9) compared to Control (43.88±1.39) (Figure 1).

**Figure 2:**
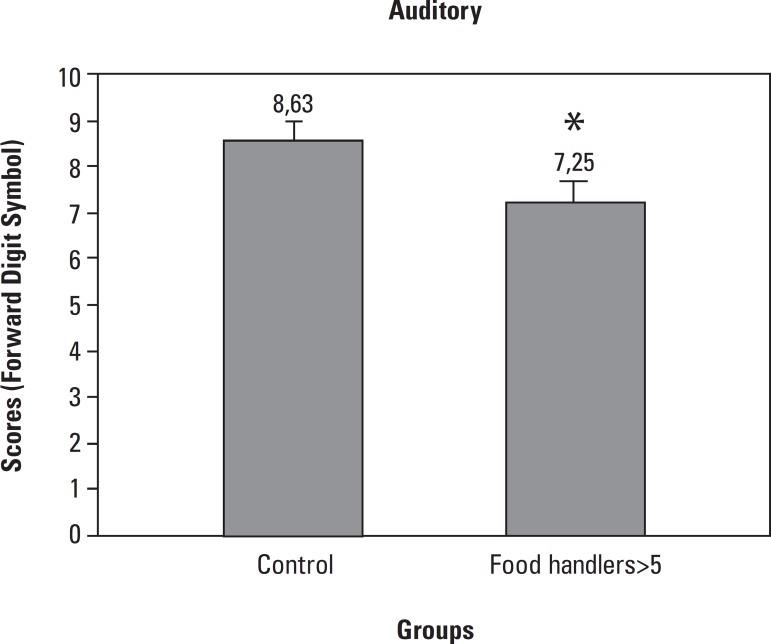
Auditory attention was evaluated by the Forward digit span test in Food Handlers with more than 5 years in the profession (FH>5, n=38) and compared to Food Handlers with less than 5 years in the profession (Control, n=29). Data analyzed by the Mann-Whitney test expressed as Median (Minimum and Maximum), p<0.05*.

In the forward digit span test, FH>5 presented lower auditory attention (7.25±0.4) compared to Control (8.63±0.38) (Figure 2).

**Figure 3:**
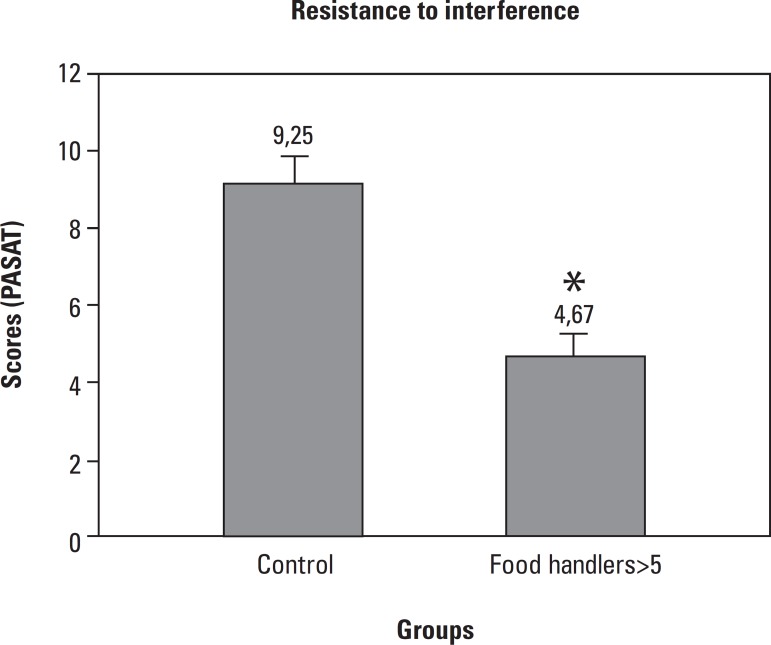
Resistance to interference was evaluated by the Paced Auditory Serial Addition Test (PASAT) test in Food Handlers with more than 5 years in the profession (FH>5, n=38) and compared to Food Handlers with less than 5 years in the profession (Control, n=29). Data analyzed by the Mann-Whitney test expressed as Median (Minimum and Maximum), p<0.05*.

FH>5 presented lower resistance to interference (4.67±1.1) compared to Control (9.25±1.16) (Figure 3).

## Discussion

The results of this study showed that food handlers with more than five years in the profession, who work for outsourced catering companies of a public hospital in Recife city, presented stress with predominance of physical symptoms in the resistance phase and also reduced focused attention, auditory attention or attention span, and lower resistance to interference capacity.

The presence of stress observed in these professionals corroborates the findings of Souza & Morais (2007).^16^ This result possibly stems from the interaction between the administration model of these catering companies and the way in which these handlers react to exposure to the rules.^7^ This reaction depends on personal variables such as personality, mood and past experiences that influence the interpretation of the factors made by the handler, causing irritation or otherwise.^6^

The interpretation of these factors allows identification of an overall relationship to personality and mood aspects, although these results found do not constitute the totality of the assessed professionals. According to Villalobos (1999 *apud* Souza & Morais, 2007), the relationship among people, the lack of identification of the employee with the administrative model of the company, the work endurance, factors related to career development are all possible predictors of stress. The author also emphasized that the responses to these stressors may be irritability, anger, dwelling on a single issue, anxiety, depression, dissatisfaction at work, disinterest, lack of motivation, sudden urge to start new projects, muscle tension, among others.^16,17^

Some authors state that there are multifactorial aspects underlying work-related diseases and draws attention to the conflicts in organizations that arise from discrepancy between forms of work organization and the need for well-being and pleasure perceived by the workers.^18^

The phenomenon created by the dialectic between employer and employee is called organizational climate.^19^

The organizational climate reflects the rules, values of the formal organization system as well as their analysis, and affects the internal and external disputes of all the types of people the organization attracts, its work processes, the modalities of communication and the exercising of authority in the organization, among other variables.^20^

This climate is not necessarily perceived in the same way by all its members, because of differences in personality, time of service, sector, function, type, the nature of activities, the organization’s forms of work plus leadership profile, opportunities, among other factors.^17^

Thus, the conflicts between the subject and their work reality may trigger processes of suffering and illness. Stress numbers amongst the main problems related to these diseases,^16^ which may affect several systems, such as the immune system, the nervous system and cognitive functions including attention.^9^

Regarding cognitive functions, attention was assessed in this study, revealing lower focus of attention and/or psychomotor speed in food handlers with more than 5 years in the profession, in contrast to findings of Ribas et al. (2010).^21^ However, it is important to clarify the methodological differences in the two studies. The present study investigated the effect of stress on Food Handlers’ attention. This group of professionals makes little use of the cognitive functions, typically carrying out manual tasks according to previous orders not involving planning. Ribas et al. (2010) on the other hand, investigated the effect of stress on air traffic controllers’ attention. In this profession, in contrast to Food Handlers’, the professionals make use of the cognitive function in complex tasks repeatedly during the work activity, involving readings, using computers, interpreting air navigation, etc.^22^

For sustained attention or concentrated attention (d2 test) and mental manipulation (Digit span in reverse order) there was no significant difference among the evaluated groups. However, the attention evaluation of auditory attention or information storage (Span of digits in direct order) revealed a significant reduction in the group of food handlers with more than 5 years in the profession compared to the control group. These results corroborate some earlier findings.^23,24^ While these studies were the only investigations in the literature involving stress and attention, it is essential to point out the difference between them, which lies in the subjects and stress expression. These latter studies were carried out in victims of sexual abuse and war veterans, all of whom had Post-traumatic Stress Disorders (PTSD) but the concordance of the results of these studies may be of some relevance, since in cases of long-term stress and PTSD, attention performance may be associated to frontal cortex dysfunction and its connections with the limbic system.^25,26^

For Food Handlers, the reduction in the auditory attention capacity or span of attention may impact information storage and affect preparing of food, recipes, orders to make minor adjustments for lack of foodstuffs, changes to the food delivery schedule, etc.

In relation to the resistance to interference capacity (PASAT test), a significant reduction among the Food Handlers with more than 5 years in the profession was detected compared to the Control group. This result points to a greater possibility of distraction during the tasks of these professionals. This implies that among food handlers there is a higher risk of mistakes during the preparation of food as well as of potential accidents, particularly where informal chatting occurs, for the subject may not resist the interference and lose their concentration. No studies were found in the literature involving stress and resistance to interference evaluations.

Further studies with a longitudinal design may contribute toward a better comprehension of the stress effects over the long term in these professionals, since the literature has indicated that traumatic events in childhood, characterized chronically, may interfere in the maturation process and cerebral organization, due to chronic hyperactivation of the neural systems of stress response.^27,28^ Future studies are envisaged which involve real control of the assessed subjects’ daily meals by applying a questionnaire or food diary, since the ingestion for example of antioxidants, formed by long-chain polyunsaturated fatty acid, water-soluble compounds such as vitamin C, beta carotene and zinc, allow the removal of the excess of reactive species of oxygen and nitrogen, thus avoiding the oxidative stress that may confound the underlying reasoning in the investigative process.^29^

Although these results are quite significant from a statistical viewpoint to this group of people studied, there are limitations in this study. In addition to the possible physiological interference during the maturation of the nervous system caused by minor traumas in childhood or nutritional aspects, there is also a form of subject and world conception formed in the personality structure acquired in the relationship with parents that may cause irritation by not identifying with the administrative model of some food companies. Future studies should focus on these subjects by using the Marilda Lipp’s quality of life inventory which can demonstrate the origin of the stress, encompassing social, emotional, occupational and health care aspects.

## Figures and Tables

**Table 3:** Phases of stress.

Subjects	Absence			Alert			Resistance			Near-exhaustion	
	Freq.	%		Freq.	%		Freq.	%		Freq.	%
Control	24	**82.76%**		0	**0%**		5	**17.24%**		0	**0%**
FH>5	10	**26.32%**		0	**0%**		28	**73.68%**		0	**0%**
Total	25			6			31			35	

Sixty-seven (67) male food handlers aged 20-35 years. The subjects were divided into 2 groups: food handlers with less than 5 years in the profession (Control, n=29) and food handlers with more than 5 years in the profession (FH>5, n=38). Statistics: Chi-square test, p>0.05.
